# Associations between Awareness of Sexually Transmitted Infections (STIs) and Prevalence of STIs among Sub-Saharan African Men and Women

**DOI:** 10.3390/tropicalmed7080147

**Published:** 2022-07-26

**Authors:** Meghana Vasudeva, Raja Nakka, Shannon Stock, Musie Ghebremichael

**Affiliations:** 1 The Ragon Institute of MGH, MIT, and Harvard, 400 Technology Square, Cambridge, MA 02139, USA; mvasudeva@mgh.harvard.edu (M.V.); rnakka@mgh.harvard.edu (R.N.); 2 Department of Mathematics, College of the Holy Cross, Worcester, MA 01610, USA; sstock@holycross.edu; 3 Department of Medicine, Harvard Medical School, 400 Technology Square, Cambridge, MA 02139, USA

**Keywords:** HIV, STIs, antiretroviral therapy, sub-Saharan Africa

## Abstract

Treatments for HIV and other STIs are not readily available in sub-Saharan Africa and other resource-limited areas, where the prevalence of HIV and other STIs is high. In the absence of treatment and laboratory infrastructure to monitor treatment efficacy, increasing awareness of STIs and STI screening are crucial components of STI prevention programs. In the current study, we sought to estimate the awareness of STIs in resource-limited countries and evaluate the strength of the association between the awareness of STIs and STIs infection. We did a secondary analysis of data obtained from 2019 women and 794 men enrolled in a community-based study that was conducted from November 2002 to March 2003 in the Moshi Urban District of Northern Tanzania. We found gonorrhea, syphilis, and HIV/AIDS were well-known among the study participants. However, their awareness of other STIs, including herpes, was very low. We also found that the awareness of STIs was not associated with STIs in men, but women who had prior knowledge of gonorrhea, syphilis, and HIV had a twofold higher risk of testing positive for an STI. Education programs aimed at increasing awareness of STIs are needed in the region. The majority of the existing STI education programs in the region focus exclusively on HIV/AIDS. The expansion of the existing AIDS/HIV education programs needs to be strengthened to include information about other STIs.

## 1. Introduction

The prevalence of sexually transmitted infections (STIs) has increased significantly in recent years, and such disorders pose a significant threat to millions of people [1,2]. Sexual contact, whether vaginal, anal, or oral, is a major source of STI transmission. The spread of STIs can also occur through nonsexual means, such as through the transfer of blood or blood products [3]. The most common STIs are chlamydia, gonorrhea, syphilis, trichomonas, mycoplasma genitalium, HIV-1, and HSV-2. According to World Health Organization and UNAIDS reports, the estimated incident cases of the most common STIs were: 127.2 million (95% UI: 95.1–165.9 million) chlamydia; 86.9 million (95% UI: 58.6–123.4 million) gonorrhea; 156.0 million (95% UI: 103.4–231.2 million) trichomoniasis; 6.3 million (95% UI: 5.5–7.1 million) syphilis; 37.7 million HIV and 491.5 million HSV type 2 cases [4,5,6]. Low- and middle-income countries (LMIC) in areas such as sub-Saharan Africa account for up to 75–85% of new STI cases [2,7,8]. Although both men and women can contract STIs, women are more vulnerable to these infections [9,10]. This may be due to the thinner and more delicate vaginal passage that is more vulnerable to infection by bacteria and viruses. Furthermore, the moist environment of the vagina makes it an ideal environment for bacteria to thrive [11]. According to the 2016 global prevalence estimates of STIs, higher rates of STIs were reported for women: trichomoniasis (5.3%), chlamydia (3.8%), gonorrhea (0.9%), syphilis (0.5%), and herpes (63.7%). The rates in men were trichomoniasis (0.6%), chlamydia (2.7%), gonorrhea (0.7%), syphilis (0.5%) and herpes (36.2%). [4,6]. If left untreated, STIs may lead to a wide range of health complications, including damage to the reproductive system and death. In men, STIs (such as gonorrhea and chlamydia) cause inflammation and painful, swollen testicles, which may lead to infertility [11,12]. The adverse effects of STIs in women include fallopian tube inflammation, infertility, abortions, stillbirths, perinatal and neonatal complications, and mother-to-child transmission [11,13]. Patients with STIs often present at least one of the following symptoms: lower abdominal pain, abnormal genital discharge, foul genital smell, excessive genital secretions, enlargement and swelling of lymph nodes in the groin area, itching in the genital area, pain during intercourse, and genital ulcers [14,15]. These symptoms are often used in resource-limited settings to detect and treat STIs [14,15]. Although the syndromic diagnosis of STIs is the most practical, feasible, and cost-effective diagnostic tool in resource-limited settings, several studies reported that it has low accuracy [14,15,16].

In sub-Saharan Africa and other resource-limited settings, despite the high prevalence of STIs, an awareness of STIs other than HIV is very low [17,18]. Many factors may contribute to this, such as limited access to health care and routine medical checkups or long periods of disease latency [13,19,20]. As a result, people do not notice that they have STIs until they experience more severe health problems [20]. Even after symptoms or disease manifestations are apparent, people in resource-limited countries may not seek medical attention. This is often due to their low access to healthcare or skepticism regarding modern medicine; many people instead rely on traditional healers, unqualified practitioners, or street vendors, who themselves are unaware of STIs [7,21,22]. Furthermore, in some of these countries, talking about sex is taboo, thereby resulting in widespread ignorance about STIs [23,24]. Due to the stigma associated with STIs, government education programs about sex and STIs often face strong opposition from the public [23].

Treatments for HIV and other STIs are not readily available in sub-Saharan Africa and other resource-limited areas, where the prevalence of HIV and other STIs is high [14,15]. In the absence of treatment and laboratory infrastructure to monitor treatment efficacy, increasing awareness of STIs and STI screening are crucial components of STI prevention programs. In the current study, we sought to estimate the awareness of STIs in resource-limited countries and evaluate the strength of the association between the awareness of STIs and the likelihood of having an STI infection. We hypothesized that a low STI awareness is associated with a higher prevalence of STIs. To test this hypothesis, we conducted a secondary analysis of data obtained from a previous cross-sectional study comprising sub-Saharan African men and women.

## 2. Materials and Methods

**Study Design and Participants**: The study included 2019 women and 794 men. The study participants were enrolled in a community-based, cross-sectional study that was conducted from November 2002 to March 2003 in the Moshi Urban District of Northern Tanzania. The institutional review boards of the Harvard School of Public Health, University of Maryland, Kilimanjaro Christian Medical Centre, and the Centers for Disease Control and Prevention approved the study protocol. The rationale, organization, and recruitment of the subjects, and the laboratory methods used have been described previously by Larsen et al. and the references therein [25].

**Study Variables**: Information on socio-demographic variables, including age, marital status, and education, was obtained. High-risk behaviors, including age at first sex, number of sexual partners, and condom use, were obtained. The participants were given a worksheet to measure STI awareness and asked to circle the disease or infection they thought could be transmitted through sexual intercourse. Symptoms of STIs, including abdominal pain, abnormal genital discharge, foul smell in the genital area, excessive genital secretions, swelling of lymph nodes in the genital area, itching in the genital area, burning pain on micturition, pain during intercourse, and genital ulcers, were recorded. Blood and urine samples were obtained from the study participants who agreed to further testing for STIs. The blood and urine samples were tested for HIV-1, HSV-2, syphilis, chlamydia, gonorrhea, Trichomonas, and Mycoplasma genitalium.

**Statistical Analysis:** Descriptive measures (such as median, IQR, percent, and frequency) and statistical graphs were used to summarize the data. Exact binomial confidence intervals were used to estimate the confidence intervals for rates of STI awareness. Fisher’s exact test and logistic regression models were used to evaluate the association between the awareness of STIs and the prevalence of STIs and STIs symptoms. The analysis was conducted using the R programming language.

## 3. Results

Table 1 presents the descriptive characteristics of the study participants. The male participants (median = 36 years; IQR: 30–43) were older than the women (median = 28 years; IQR 24–35). The men and women had a median age at first sex of 18 years (IQR 16–20) and 19 years (IQR 17–21), respectively. The majority of the men and women had pre-secondary school education. Thirty percent (30%) and 27% of the men and women reported post-secondary school education, respectively. Sixty percent (60%) of the women were married and stayed with a single partner, while 99% of the men confirmed that they were either married or living with the interviewed women. Eighty-eight percent (88%) of men and 77% of women had one partner 12 months prior to the interview. Moreover, 97% of the men and 23% of the women had been circumcised. Most of the men and women were long-term residents of the Moshi Urban District. Seventy-nine percent (79%) of the men and 64% of the women had never used a condom.

**Figure 1:**
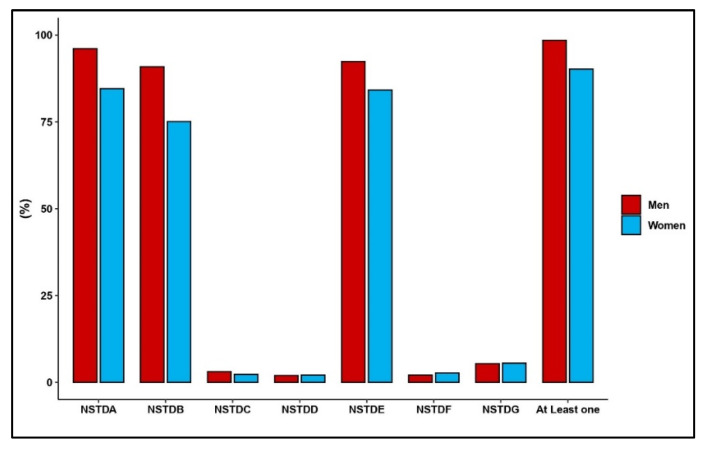
Prevalence of self-reported STI knowledge among men and women in Moshi, Tanzania, 2002–2003: (NSTDA: Gonorrhea, NSTDB: Syphilis, NSTDC: Chlamydia, NSTDD: Genital Ulcers, NSTDE: AIDS, NSTDF: Herpes, NSTDG: Others).

Figure 1 displays the prevalence rates of STI awareness among the study participants. Ninety-nine percent (*n* = 782) of the men and 91% (*n* = 1827) of the women reported that they had prior knowledge of at least one of the STIs. Most of the men had an awareness of gonorrhea (96%; *n* = 763), AIDS (92%; *n* = 734), and syphilis (91%; *n* = 722). As was the case with men, most of the women had an awareness of gonorrhea (85%; *n* = 1708), HIV/AIDS (84%; *n* = 1701), and syphilis (75%; *n* = 1517). The awareness rates of other STIs among men and women were less than 5%.

**Figure 2:**
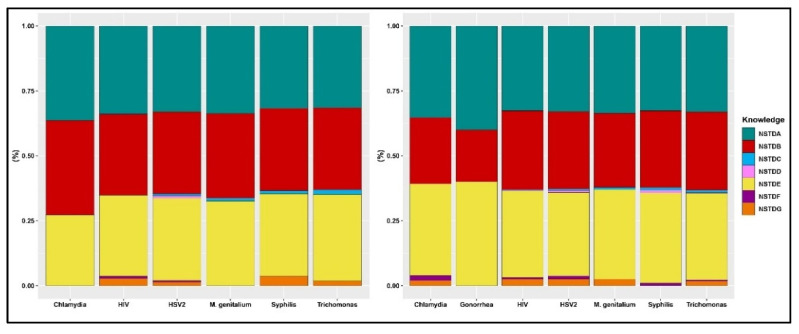
Prevalence of self-reported STI knowledge among men (**left**) and women (**right**) who tested positive for STIs. (NSTDA: Gonorrhea, NSTDB: Syphilis, NSTDC: Chlamydia, NSTDD: Genital Ulcers, NSTDE: AIDS, NSTDF: Herpes, NSTDG: Others).

**Figure 3:**
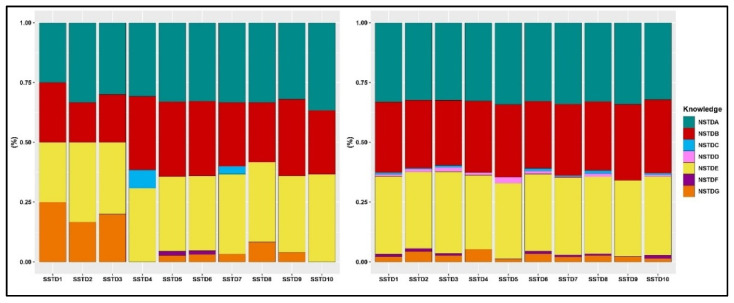
Prevalence of self-reported STI knowledge among men (**left**) and women (**right**) who experienced symptoms. (Symptoms—SSTD1: abdominal pain, SSTD2: abnormal genital discharge, SSTD3: foul smell in the genital area, SSTD4: excessive genital secretions, SSTD5: swelling of lymph nodes in the genital area, SSTD6: itching in the genital area, SSTD7: burning pain on micturition, SSTD8: pain during intercourse, SSTD9: genital ulcers, and SSTD10: others). (Knowledge—NSTDA: Gonorrhea, NSTDB: Syphilis, NSTDC: Chlamydia, NSTDD: Genital Ulcers, NSTDE: AIDS, NSTDF: Herpes, NSTDG: Others).

The prevalence rates of STI awareness among men and women who tested positive for each STI are presented in Figure 2. Gonorrhea, syphilis, and HIV/AIDS were well-known among both men and women who tested positive for any STI. The awareness rate for each of these STIs ranged from 27–36% among men who tested positive for STIs. In women who tested positive for STIs, the awareness rate of these three STIs ranged from 20–40%. In both men and women who tested positive for STIs, the awareness rate of the other STIs was less than 5%. Figure 3 displays the prevalence rates of STI awareness among men and women who reported STI symptoms. Similarly, gonorrhea, syphilis, and HIV/AIDS were well-known among both women and men who reported STI symptoms. As was the case with those who tested positive for STIs, men and women who reported STI symptoms were less aware of the other STIs except gonorrhea, syphilis, and HIV/AIDS.

**Figure 4:**
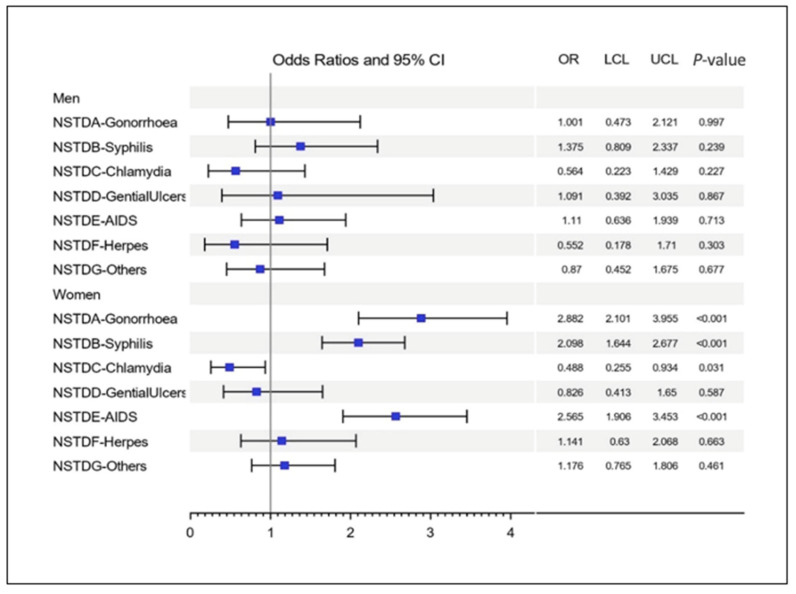
Forest plot presenting odds of testing positive for at least one STI in men and women who reported awareness of an STI.

We evaluated the association between the awareness of STIs and STI infection and symptoms. An awareness of STIs was not associated with STI symptoms in both men and women (data not shown). Figure 4 displays the odds of testing positive for at least one STI. An awareness of STIs was not associated with testing positive for STIs in men. However, in women, the odds of testing positive for an STI were associated with an awareness of STIs. Interestingly, women who were aware of gonorrhea (OR = 2.88, 97.5% CI = 2.1–3.9, *p* < 0.01), syphilis (OR = 2.09, 97.5% CI = 1.6–2.6, *p* < 0.01) and HIV/AIDS (OR = 2.56, 97.5% CI = 1.9–3.4, *p* < 0.01) were more likely to test positive for STIs. Women who had prior knowledge of chlamydia were 51% less likely to get tested for STIs compared to women who were not aware of chlamydia (OR = 0.49; 95% CI: 0.255–0.934; *p*-value = 0.0304). Most of the significant results found were contrary to what we hypothesized.

## 4. Discussion

This paper aimed to estimate the prevalence of STI awareness and evaluate their association with STI infection status, defined by both syndromic and laboratory testing. Our results showed that STI awareness was highest among men and women for the following infections: gonorrhea (96% among men, 85% among women), HIV (92% among men, 84% among women), and syphilis (91% among men, 75% among women). Interestingly, active gonorrhea infection was uncommon within our study population, with just 0.1% (*n* = 2) of women infected and no men [14,15]. It is not surprising that most study participants knew about HIV/AIDS, as the disease was present at relatively high rates among both men and women (men: 7%, women: 11%). Herpes was least known (men: 2.1%, women: 2.7%) but had the highest prevalence among all STIs in both men and women (men: 39%, women 43%) [14,15]. Awareness of syphilis, gonorrhea, and HIV/AIDS was higher among men and women who tested positive for these infections and other STIs.

Most of the participants in this study knew about STIs. Ninety-nine percent of men and 91% of women were aware of at least one STI. This finding is consistent with findings from other studies [26,27,28,29]. Moreover, the study participants demonstrated a higher knowledge of gonorrhea, syphilis, and HIV/AIDS. This finding is also consistent with the results of previous studies, which reported a higher awareness of STIs caused by bacteria (such as syphilis and gonorrhea) than by viruses [30]. Due to the high prevalence of HIV in the region and the extensive media attention given to HIV/AIDS in the past three decades, a higher awareness of HIV/AIDS among the study participants was expected. However, awareness of herpes was quite low, despite the higher prevalence of herpes in the region. Other studies from the region also reported a lower awareness of herpes. For example, a study in Nigeria reported that only 10% of the study participants knew about herpes [27]. Herpes is sometimes called a hidden STI and resides in the body in the latency stage for years [20]. This could be the reason why awareness of it in our study or other related studies was low.

We found that awareness of STIs was not associated with STI symptoms and testing positive for STIs in men. A study by Nsuami et al. reported a similar finding [31]. The study reported a lack of associations between having knowledge of STIs and infections with chlamydia and gonorrhea. Moreover, we found awareness of STIs was not associated with STI symptoms in women. However, our study revealed that women who had prior knowledge of gonorrhea, syphilis, and HIV had a twofold higher risk of testing positive for an STI. Higher associations between a higher risk of STIs and STI knowledge were also reported by others [32,33,34,35,36]. Interestingly, women who had prior knowledge of chlamydia were 51% less likely to test positive for STIs compared to women who were not aware of chlamydia. Due to the cross-sectional nature of the study, we were unable to ascertain if study participants knew about STIs after becoming infected. This might be the reason for the unexpected result of higher STIs among the study participants who had more knowledge of STIs. As others have reported, knowledge about STIs was most likely acquired from direct experience than from learning how to prevent infection [32]. Nzoputam et al. reported that respondents with a good knowledge of STIs had a greater tendency to test positive for STIs [33].

Our study has an advantage over previous related studies: it is a population-based study with large sample sizes. As opposed to previous related studies, we did not study specific groups, such as STI clinic attendees or sex workers. Furthermore, our data on STI status came from both biologically confirmed tests and self-reported symptoms. However, the study has some limitations. It was a cross-sectional study; hence, we were unable to ascertain if the study participants knew about STIs after becoming infected. Men in the study were partners/spouses of women. Thus, the population of men is not representative of men in general, but rather sexual partners of the women. Moreover, only men and women who consented to be tested for STIs and those willing to answer questions on their awareness of STIs were included in the analysis. Further, the information provided by the study participants on STI symptoms and awareness was self-reported, which might have resulted in under-reporting. In this region, STI infection, symptoms, and knowledge often result in a negative reaction among the community. Thus, self-reported sexual behaviors, STI awareness, and STI symptoms might have been underestimated due to social desirability bias.

## 5. Conclusions

The findings of this study highlight a limited awareness of some STIs in sub-Saharan Africa, where HIV/AIDS and other STIs are highly prevalent. In this region, STI treatments are not readily available, or there is an inadequate laboratory infrastructure to monitor treatment efficacy [14,15]. Education programs aimed at increasing awareness of STIs are needed in the region. Increasing awareness about STIs should be a crucial component of STI prevention programs [17,18]. The majority of the existing STI education programs in the region focus exclusively on HIV/AIDS. The expansion of the existing AIDS/HIV education programs needs to be strengthened to include information about other STIs. Raising awareness of STIs will help people identify the symptoms of STIs and seek medical care. Many people in the region turn to informal treatments, including visiting traditional healers [22]. Given that they are alternative care providers, traditional doctors or healers should also be targeted by awareness-raising education programs.

## Figures and Tables

**Table 1:** Demographic characteristics of men (*n* = 794) and women (*n* = 2019) in Moshi, Tanzania, 2002–2003.

Variable		Men Median	IQR	Women Median	IQR
Age (years)		36	30–43	28	24–35
Age (years) at First Sex		18	16–20	19	17–21
		**N**	**%**	**N**	**%**
Marital Status					
	Currently Married	784	98.7	1218	60
	Never Married	10	1.3	800	40
Stay *					
	≥10 years	537	67.6	1036	51
	<10 years	256	32.2	981	49
Education					
	Pre-High School	550	69.3	1483	73.5
	High School	234	29.5	536	26.5
Circumcision					
	Yes	768	96.7	459	23
	No	25	3.1	1556	77
Condom Use					
	Never	630	79.3	1296	64.2
	Sometimes	115	14.5	353	17.5
	Often/Always	47	5.9	43	2.1
Number of Partners 12 months					
	1	695	87.5	1553	76.9
	2+	98	12.4	44	2.2

* Stayed/Lived in Moshi District.

## Data Availability

The dataset used in the manuscript is available from the corresponding author upon reasonable request.
